# Teacher-Reported Prevalence of FASD in Kindergarten in Canada: Association with Child Development and Problems at Home

**DOI:** 10.1007/s10803-020-04545-w

**Published:** 2020-05-29

**Authors:** Jacqueline Pei, Caroline Reid-Westoby, Ayesha Siddiqua, Yomna Elshamy, Devyn Rorem, Teresa Bennett, Catherine Birken, Rob Coplan, Eric Duku, Mark A. Ferro, Barry Forer, Stelios Georgiades, Jan Willem Gorter, Martin Guhn, Jonathon Maguire, Heather Manson, Rob Santos, Marni Brownell, Magdalena Janus

**Affiliations:** 1 Department of Educational Psychology, University of Alberta, 6-131 Education North, Edmonton, AB T6G 2G5 Canada; 2 Offord Centre for Child Studies, Department of Psychiatry and Behavioural Neuroscience, McMaster University, Hamilton, ON Canada; 3 Department of Health Research Methods, Evidence, and Impact, McMaster University, Hamilton, ON Canada; 4 Department of Pediatrics, University of Toronto, Toronto, ON Canada; 5 The Hospital for Sick Children, Toronto, ON Canada; 6 Department of Psychology, Carleton University, Ottawa, ON Canada; 7 School of Public Health and Health Systems, University of Waterloo, Waterloo, ON Canada; 8 Human Early Learning Partnership, School of Population and Public Health, University of British Columbia, Vancouver, BC Canada; 9 Department of Pediatrics, McMaster University, Hamilton, ON Canada; 10 CanChild Centre for Childhood Disability Research, McMaster University, Hamilton, ON Canada; 11 Li Ka Shing Knowledge Institute, St. Michael’s Hospital, Toronto, ON Canada; 12 Public Health Ontario, Toronto, ON Canada; 13 Community Health Services, University of Manitoba, Winnipeg, MB Canada; 14 Manitoba Centre for Health Policy, Department of Community Health Sciences, University of Manitoba, Winnipeg, MB Canada

**Keywords:** Fetal Alcohol Spectrum Disorder, Prevalence, Problems at home, Early Development Instrument, Developmental health, Kindergarten

## Abstract

The prevalence of Fetal Alcohol Spectrum Disorder (FASD) may be underestimated as it can be difficult to diagnose in early childhood possibly reflecting unique developmental trajectories relative to other Neurodevelopmental Disabilities (NDDs). Using data collected via the Early Development Instrument (EDI) between 2010 and 2015, we examined the prevalence of kindergarten children with FASD and their concurrent developmental outcomes. We found that the prevalence of FASD ranged from 0.01 to 0.31%. A greater percentage of children with FASD had teacher-reported home problems that interfered with their classroom functioning. Overall children with FASD had higher mean scores on the developmental domains compared to children with NDDs. Results of the current study can inform strategies and policies for early identification and intervention.

**Electronic supplementary material:**

The online version of this article (10.1007/s10803-020-04545-w) contains supplementary material, which is available to authorized users.

Fetal Alcohol Spectrum Disorder (FASD) involves physical, mental, and behavioral deficits due to prenatal alcohol exposure (PAE; Cook et al. 2016). FASD falls into the category of neurodevelopmental disorders (NDDs), a group of conditions with similar cognitive and behavioural processes, such as difficulties in learning, language, motor skills, behaviour, and neuropsychological functions (Alabaf et al. 2019; Dewey 2018; Popova et al. 2019). NDDs include Autism Spectrum Disorder (ASD; Geschwind and Levitt 2007), Attention Deficit Hyperactivity Disorder (ADHD; Ehninger et al. 2008), and Intellectual Disability (ID; Dewey 2018). Individuals with FASD have been identified as being highly diverse in their presentation, having a variety of possible impairments in many areas of functioning including cognition (Davis et al. 2013), learning (Rasmussen and Bisanz 2011), language (Wyper and Rasmussen 2011), attention (Kooistra et al. 2011), memory (Pei et al. 2011b, b), executive functioning (Rasmussen and Bisanz 2009), motor abilities (Bay and Kesmodel 2011), and affect regulation (Pei et al. 2011a). The estimated prevalence of FASD in Canada is 4.4% based on combined studies (Flannigan et al. 2018), which parallels prevalence rates in the United States (US) that range from 1.1 to 5% (May et al. 2018).

Researchers have suggested that the global prevalence of FASD among elementary school students may be underestimated (Popova et al. 2019). FASD (which includes Fetal Alcohol Syndrome (FAS) and Partial FAS) can be difficult to diagnose in early and middle childhood for many reasons. Challenges include the high level of heterogeneity in clinical presentation of the FASD population, and the difficulty detecting impairments across multiple domains of functioning at an early age. Also, many features impacted by FASD, such as executive functioning and verbal reasoning are not seen in typically developing children until at least 12 years of age (Rasmussen 2005; Rasmussen and Bisanz 2009). Additionally, there are reports of high rates of comorbidities in children with FASD. For instance, overlapping symptoms between FASD and Attention Deficit Hyperactivity Disorder (ADHD; Brown et al. 2012) and FASD and Autism Spectrum Disorder (ASD; Helgesson et al. 2018), have been documented. Yet, it is crucial to understand the early presentation and distinguishing components of FASD in order to have networks in place prior to school entry to optimize current supports, alert educators and families to future assistance that may be warranted, provide information to facilitate long-term planning, and ensure access to funding, as appropriate. Combining these supports set children with FASD up for success in learning and other aspects of their life and development. Developing analytical methods that distinguish FASD characteristics at a young age can contribute to more effective and timely diagnostic approaches and early intervention initiatives.

Early detection of FASD, as with other disabilities, has been associated with reduced risk for adverse outcomes (Streissguth et al. 1996, 2004). In general, detection is crucial prior to entering school as this influences the accommodation and special education opportunities, that helps to create a healthy educational trajectory of a child, as well as affecting health into adulthood (Janus 2011). Although some NDDs are more commonly identified in preschool, there is a lack of a clear understanding about certain NDDs, such as FASD, especially regarding their presentation or unique characteristics early in life. This is due in large part to limitations in neuropsychological assessment measures and the range of adverse environmental factors that impact development such as multiple foster placements and history of abuse (Benz et al. 2009; Marschik et al. 2017; May et al. 2018).

## Identifying FASD

The term Fetal Alcohol Syndrome (FAS) was first coined in (Jones and Smith 1973), making it a relative newcomer to the field of NDDs. FAS falls under the broad diagnostic term of FASD, which includes all intellectual and developmental challenges due to prenatal alcohol exposure (Blackburn and Whitehurst 2010). When compared to more well-known neurodevelopmental disorders such as ASD (Mazurek et al. 2017), the criteria for making a diagnosis of FASD is still in its early stages. In Canada, the first national diagnostic guidelines were published in 2005, which recommended a multidisciplinary approach that incorporated the Institute of Medicine (IOM) classification and the 4-digit diagnostic code system into a single model (Astley and Clarren 2000; Chudley et al. 2005). Within this diagnostic framework, individuals were assessed in four areas: growth deficiency, abnormal facial development, central nervous system or brain impairment, and prenatal exposure to alcohol (Chudley et al. 2005) with consideration of other prenatal risk factors (e.g., prenatal care and complications, genetic risk factors, and in utero exposure to other teratogens), as well as postnatal risk factors (e.g., abuse, disrupted living arrangements, head injuries, and exposure to violence; Benz et al. 2009; Chudley et al. 2005; Marschik et al. 2017). Recently, these guidelines were reviewed and revised to reflect ongoing research in the field (Cook et al. 2016). Changes included new diagnostic terms such as FASD, modifying and clarifying domains of impairment, removal of growth deficiency criteria, and inclusion of an ‘at risk’ designation for young children who might have prenatal alcohol exposure but fail to meet criteria for a diagnosis (Cook et al. 2016).

Identifying and diagnosing FASD in young children is complicated by the high rates of early life adversity noted for this population (Streissguth et al. 1996, 2004). Experiencing early life adversities, such as exposure to abuse, neglect, and household dysfunction tends to predict poor physical and mental health outcomes in the general population (Dube et al. 2003). The deleterious neurodevelopmental outcomes associated with early life adversity tends to be more severe for children with prenatal alcohol exposure (Henry et al. 2007). Thus, the presence of these factors may increase the difficulty of detection of FASD, as well as elevate vulnerability for this population—underscoring the importance of early detection. For example, compared to patients with FASD from stable home environments, patients with FASD who live in unstable homes are at a greater risk for disrupted school experiences, trouble with the law, and alcohol and drug problems (Streissguth et al. 2004). Early identification creates opportunities for early intervention that may mitigate these risks.

Thus far, our knowledge of the prevalence of FASD among kindergarten children across provinces and territories in Canada, or indeed other countries, remains limited. Despite their greater susceptibility, the prevalence of children with FASD who experience early life adversities also remains unknown. Examining the differences in developmental health of children with FASD and other NDDs has particular value, as this may assist educators in planning interventions for children who are most likely to benefit. Combined with information on the prevalence of FASD and the proportion of them who experience early life adversities, a better understanding of their developmental health (broadly understood as age-appropriate status in all domains of development; Keating and Hertzman 1999) across jurisdictions is necessary for informing strategies in a pragmatic manner. This includes implementing strategies for early identification as well as addressing community resources, providing necessary early intervention services in areas of need.

Using data collected via the Early Development Instrument (EDI; Janus and Offord 2007), a teacher-completed measure of children’s development at school entry, the primary objectives of this study were to determine, among kindergarten children across Canadian provinces and territories, (1) the prevalence of a teacher-reported diagnosis of FASD, and (2) the concurrent developmental health status of children with FASD across Canada and in a few provinces with sufficient numbers. A secondary objective of this study was to identify the prevalence of problems at home among these children in comparison with children with other developmental disabilities. It was not the goal of this study to investigate the difference in developmental status between children with FASD and those without any neurodevelopmental disorders. Understanding the geographic distribution of young children diagnosed with FASD across Canada through the examination of educator-reported diagnoses, may provide insight into regional disparities in diagnostic practices prior to the introduction of the new guidelines. By exploring the areas of identified disability and family/environment factors present for this population in comparison to other developmental disabilities, we attempt to establish contextual-level characteristics that may be unique to children with FASD.

## Methods

### Study Population

Data for this paper come from the Canadian Children’s Health in Context Study (CCHICS; Janus et al. 2018, 2019), a population-wide database designed to monitor the developmental health of children with health disorders across Canada. The study used EDI data collected from 2010 to 2015 on 603,904 children attending kindergarten in publicly-funded schools across Canada. There were multiple implementations of the EDI in some provinces and territories over time during this study period (Janus et al. 2018). New Brunswick, Prince Edward Island, and Nunavut were excluded from the study sample, as EDI data were not collected in these provinces during the study period. In total, teachers reported 658 children with a physician or psychologist diagnosis of FASD and 8465 with a diagnosis of NDD (this group includes ASD, Global Delay, Down Syndrome, Intellectual Delay, Rett’s Disorder, and Learning Disorders). Children were included in the study if they had a valid EDI questionnaire (no more than 25% of missing items), if they were in their current classroom for at least a month, and if they were enrolled in kindergarten.

### Measures

#### Early Development Instrument

Data on children’s developmental health in kindergarten were collected with the EDI (Janus and Offord 2007), a 103-item, teacher-completed checklist. The EDI has been administered at the population level in most Canadian provinces and territories since 2004. The EDI is completed by teachers for children aged 4–6 years in the second half of the school year and covers five broad domains of developmental health: physical health and well-being, social competence, emotional maturity, language and cognitive development, and communication skills and general knowledge. The five EDI domains are further divided into 16 subdomains (see Janus and Offord 2007). For the present study, children’s mean domain and subdomain scores (all on a scale from 0 to 10, where 0 indicates low ability and 10 indicates high ability) were used as the outcome variables in the analyses.

In addition to information on children’s developmental health, the EDI also includes a number of demographic questions, including child’s age at the time of EDI completion, sex, first language, and whether they have English or French as a second language (E/FSL), which indicates a child’s level of fluency in the school’s language of instruction. The EDI also collects information on children’s postal codes. Additionally, the EDI includes teacher-reported problems that can influence a child’s ability to function in a regular classroom. Children were classified as having problems at home if their teacher answered “yes” to the option “home environment/ problems at home” in response to the question: “Does the child have a problem that influences his/her ability to do school work in a regular classroom?” Teachers responded to this question based on either information from parents/guardians, information found in the students’ school records, and/or their own observations in the classroom, as instructed to do in the training. The option of “home environment/problems at home”, introduced into the EDI in 2010, was based on narrative comments teachers provided in preceding years (2004–2009), summarizing a range of issues such as family break-up, financial problem, addiction, foster care, etc. Earlier studies (Forer and Zumbo 2011; Guhn et al. 2007) found no evidence of teacher bias on the EDI responses, although this particular option was not part of the questionnaire at that time.

The EDI also includes up to three health diagnoses the child may have received by a doctor or psychological professional (see Janus et al. 2018). These items have been included on the EDI since the 2009/2010 school year and include a list of over 30 most frequent childhood diagnoses, including FASD (Janus et al. 2018). For the purpose of this study, three diagnostic groups were created: children with only a diagnosis of FASD (FASD only), children with FASD and at least one other diagnosis (FASD+), and children with a NDD other than FASD, which could be ASD, Global Delay, Down Syndrome, Intellectual Delay, Rett’s Disorder, or Learning Disorders. Children with FASD were divided into two groups based on the presence of a comorbidity, as no previous research has examined differences on the EDI domain scores specifically in this population. Also, children with NDDs were selected as a comparison group, as we felt it would be a more meaningful comparison than typically developing children, who tend to have higher scores than children with special health care needs (Goldfeld et al. 2015; Janus 2011).

The psychometric properties and validity of the EDI have been reported in many studies, as well as associations with other developmental outcomes (e.g., Forer and Zumbo 2011; Guhn et al. 2016; Janus et al. 2007; Janus and Offord 2007). Most specifically in relevance to this study, the EDI has been recently validated for the population of children with special needs (Janus et al. 2019). In the current study, the internal consistency for each of the domains, measured by Cronbach’s alpha, were as follows: 0.79 for physical health and well-being, 0.96 for social competence, 0.93 for emotional maturity, 0.92 for language and cognitive development, and 0.94 for communication skills and general knowledge.

#### Neighbourhood SES Index

Discrete neighbourhoods were created to analyze the EDI data, using a detailed set of criteria (Guhn et al. 2016). A developmentally-sensitive neighbourhood-level SES index was derived from the 2011 Canadian National Household Survey and the 2010 Income Taxfiler database, which are national surveys collected by Statistics Canada (Forer et al. 2019). This information was available for dissemination blocks (DBs), the smallest geographic areas which are equal to a city block bounded by intersecting streets (see Guhn et al. 2016 for more information about the creation of neighbourhoods). A small subset of the National Household Survey and Taxfiler variables was identified to create the neighbourhood SES index. This index consists of 10 variables that represent an optimal compromise between maximizing the variance explained in the developmental health outcomes measured using the EDI, and restricting the number of variables to a reasonable number for interpretation and intervention (see Forer et al. 2019 for a full description). The SES index was transformed into Z-scores, with a mean of 0 and a standard deviation of one. The neighbourhood-SES index was merged with the EDI dataset using children’s postal codes with a 99.3% match rate.

### Analytical Plan

Descriptive statistics, including frequencies, percentages, means and standard deviations, were used to examine the prevalence of FASD-only, FASD+, and other NDDs, as well as demographic characteristics (e.g. mean age, percent males, etc.) and the presence of home problems among children in each of these three diagnostic groups. Following this, one-way multivariate analyses of covariance (MANCOVAs) were used to determine whether there were significant differences in the five developmental domain scores of the EDI between children in the above-mentioned groups in Canada and in various jurisdictions across the country. Since Canada’s healthcare system is governed at the provincial/territorial level, we were interested in seeing if there might be differences in the developmental health of children with FASD depending on where they live. Although the diagnostic guidelines are uniform across the country (Cook et al. 2016), provinces have different paths to the identification and diagnosis of FASD, therefore, differences in these children’s developmental health may reflect these system level differences in service delivery. These analyses controlled for children’s age, sex, whether a child had English or French as a second language (E/FSL status), home problems, and the neighbourhood SES Index. Post hoc analyses were adjusted using a Bonferroni correction to identify whether children from the three diagnostic categories differed from one another.

## Results

### Demographics

Table 1 shows the demographic characteristics of children with FASD-only, FASD+, and other NDDs. In total, teachers reported 658 children with diagnosis of FASD by a doctor or psychological professional (523 with only FASD-only and 135 with FASD+) and 8,465 with a diagnosis of an NDD (this group includes children with any of the following disorders: ASD, Global Delay, Down Syndrome, Intellectual Delay, Rett’s Disorder, and Learning Disorders). Children in the FASD-only group were slightly younger compared with the other two groups (*F*_2, 9110_ = 5.54, *p* = 0.004), but it wasn’t meaningfully different. In each of these three diagnostic groups, there were more males than females, with the highest proportion being in the NDD group [χ^2^ (2, N = 9118) = 37.55, *p* < 0.001]. The greatest proportion of children with E/FSL was also observed in the NDD group [χ^2^ (2, N = 9096) = 23.68, *p* < 0.001] and the average neighbourhood-SES index z-score was highest in the NDD group (*F*_2, 9060_ = 33.95, *p* < 0.001).

**Table 1:** Demographic characteristics of children with FASD only, children with FASD and other comorbidities, and children with other neurodevelopmental disorders (NDDs)

	FASD only (n = 523)	FASD+ (n = 135)	NDDs (n = 8465)
Mean age (SD)	5.73 (0.41)	5.80 (0.40)	5.80 (0.42)
n (%) males	340 (65)	92 (68.1)	6450 (76.2)
n (%) E/FSL	29 (5.6)	6 (4.5)	984 (11.6)
Mean neighbourhood SES Index z-Score (SD)	− 0.25 (0.90)	− 0.43 (1.07)	0.04 (1.02)

NDD group includes ASD, global delay, down syndrome, intellectual delay, Rett’s disorder, learning disorders

*E/FSL* English/French as a second language

### Prevalence of FASD and Other NDDs

Table 2 displays the prevalence of children with FASD-only, FASD+, and other NDDs. Prevalence rates ranged from 0.01% (in Quebec) to 0.22% (in British Columbia) for FASD-only, 0.01% (in Ontario and Alberta) to 0.10% (in Manitoba) for FASD+, and 0.61% (in the Northwest Territories) to 1.92% (in Newfoundland and Labrador) for other NDDs.

**Table 2:** Prevalence of children with FASD only, children with FASD and other comorbidities, children with other NDDs

	FASD		FASD+		NDDs	
	n	%	n	%	n	%
Ontario	97	0.04	30	0.01	4260	1.61
Manitoba	81	0.20	41	0.10	576	1.45
Alberta	77	0.10	4	0.01	679	0.87
British Columbia	200	0.22	42	0.05	1356	1.49
Saskatchewan	31	0.13	6	0.03	196	0.85
Northwest Territories	2	0.08	1	0.04	16	0.61
Newfoundland and Labrador	11	0.08	3	0.02	257	1.92
Nova Scotia	13	0.05	8	0.03	443	1.83
Yukon	3	0.20	–	–	18	1.22
Quebec	8	0.01	–	–	664	1.01
Canada	523	0.09	135	0.02	8465	1.40

NDD group includes ASD, global delay, down syndrome, intellectual delay, Rett’s disorder, and learning disorders

### Prevalence of Home Problems

There were higher proportions of children reported as having home problems in the FASD-only (25.6%) and FASD+ (32.6%) groups than in the NDD group (11.4%). This pattern was consistent in all jurisdictions (Table 3), however, very small frequencies of children in the FASD groups make this result difficult to interpret in Alberta, Newfoundland, and Yukon.

**Table 3:** Distribution of home problems among children with FASD only, children with FASD and other comorbidities, children with other NDDs

	FASD		FASD+		NDDs	
	n	%	n	%	n	%
Ontario	33	34	11	36.7	458	10.8
Manitoba	16	19.8	11	26.8	76	13.2
Alberta	15	19.5	2	50	80	11.8
British Columbia	53	26.5	15	35.7	175	12.9
Saskatchewan	8	25.8	2	33.3	26	13.3
Newfoundland and Labrador	1	9.1	0	0	14	5.4
Nova Scotia	4	30.8	3	37.5	42	9.5
Yukon	1	33.3	0	0	4	22.2
Quebec	3	37.5	0	0	92	13.9
Canada	134	25.6	44	32.6	967	11.4

The Northwest Territories have been excluded as the home problems question was not included in their version of the EDI

### Developmental Health

The MANCOVA analysis of the five EDI domain scores for the three study groups in the full Canadian sample showed group differences on all the developmental domains (*F*_10, 17496_ = 21.92, *p* < 0.001; Pillai’s trace = 0.025) and subdomains. In order to provide some context, mean domain scores for the full sample can be found in Online Appendix 1. The univariate analyses revealed significant group differences on all the EDI domain scores (Table 4). Pairwise comparisons based on estimated marginal means and using Bonferroni adjustment for multiple comparisons revealed that children with FASD-only scored better than those with other NDDs. Furthermore, the FASD-only group scored higher than the FASD+ group on the social competence domain and the FASD+ group scored higher than the NDD one on communication skills and general knowledge.

**Table 4:** MANCOVA univariate results examining differences in scores on all five domains of the EDI in Canada among children with FASD only, children with FASD and other comorbidities, and children with other NDDs

		Mean	Standard error	F	*p*	Partial ƞ^2^
Physical health and well-being	FASD only	7.22	0.09	F (2, 180.11) = 42.50	< .001	.010
	FASD+	6.89	0.18			
	NDDs	6.38	0.02			
Social competence	FASD only	5.55	0.10	F (2, 233,41) = 42.93	< .001	.010
	FASD+	5.00	0.20			
	NDDs	4.58	0.03			
Emotional maturity	FASD only	5.81	0.07	F (2, 33.44) = 12.92	< .001	.003
	FASD+	5.60	0.14			
	NDDs	5.44	0.02			
Language and cognitive development	FASD only	6.30	0.14	F (2, 134.90) = 14.41	< .001	.003
	FASD+	6.08	0.27			
	NDDs	5.58	0.03			
Communication skills and general knowledge	FASD only	4.82	0.13	F (2, 716.79) = 87.24	< .001	.020
	FASD+	4.23	0.25			
	NDDs	3.14	0.03			

MANCOVA controlled for age, sex, EFSL status, home problems, and neighbourhood SES. Higher scores represent greater ability

Next, MANCOVAs were repeated for each jurisdiction with sufficient numbers, also examining differences in scores on the five EDI domains: Ontario, Manitoba, Alberta, and British Columbia. In Ontario, the MANCOVA examining differences on all five domains between children with FASD-only, FASD+, and other NDDs revealed significant group differences (*F*_10, 8392_ = 7.98, *p* < 0.001; Pillai’s trace = 0.019). The univariate analyses showed significant group differences on all the EDI domain scores except for emotional maturity (Table 5). Pairwise comparisons demonstrated that children with FASD-only scored significantly higher in these domains compared to children with other NDDs. The FASD+ group fell somewhere in the middle.

**Table 5:** MANCOVA univariate results examining differences in scores on all five domains of the EDI in Ontario, Manitoba, Alberta, and British Columbia among children with FASD only, children with FASD and other comorbidities, and children with other NDDs

		Ontario			Manitoba			Alberta			BC		
		Mean (SE)	F (df, mean^2^)	Partial ƞ^2^	Mean (SE)	F (df, mean^2^)	Partial ƞ^2^	Mean (SE)	F (df, mean^2^)	Partial ƞ^2^	Mean (SE)	F (df, mean^2^)	Partial ƞ^2^
Physical health and well-being	FASD only	7.45 (.21)	13.66 (2, 57.06)**	.006	7.33 (.23)	15.34 (2, 65.78)**	.04	7.72 (.23)	16.75 (2, 68.47)**	.04	6.86 (.15)	23.70 (2, 98.69)**	.03
	FASD+	7.37 (.39)			6.61 (.34)			7.65 (1.02)			6.88 (.32)		
	NDDs	6.45 (.03)			5.98 (.09)			6.31 (.08)			5.85 (.06)		
Social competence	FASD only	5.45 (.24)	8.89 (2, 46.19)**	.004	5.64 (.26)	11.12 (2, 55.84)**	.03	5.98 (.28)	11.34 (2, 64.52)**	.03	5.32 (.17)	14.27 (2, 75.88)**	.02
	FASD+	4.84 (.43)			5.02 (.37)			5.43 (1.20)			5.15 (.36)		
	NDDs	4.45 (.04)			4.40 (.10)			4.59 (.09)			4.40 (.07)		
Emotional maturity	FASD only	5.71 (.17)	2.37 (2, 6.23)	.001	5.84 (.19)	4.44 (2, 12.39)*	.01	6.22 (.18)	7.14 (2, 16.99)**	.02	5.60 (.12)	1.79 (2, 4.70)	.002
	FASD+	5.63 (.31)			5.70 (.27)			6.65 (.78)			5.36 (.25)		
	NDDs	5.36 (.03)			5.29 (.07)			5.55 (.06)			5.36 (.05)		
Language and cognitive development	FASD only	6.98 (.32)	8.29 (2, 78.82)**	.004	5.78 (.33)	4.17 (2, 35.78)	.01	6.31 (.35)	3.24 (2, 29.77)	.01	6.17 (.23)	11.22 (2, 108.90)**	.02
	FASD+	6.56 (.59)			4.96 (.48)			7.04 (1.52)			6.78 (.49)		
	NDDs	5.72 (.05)			4.76 (.13)			5.44 (.12)			5.24 (.09)		
Communication skills and general knowledge	FASD only	5.18 (.29)	35.04 (2, 266.03)**	.016	4.29 (.31)	11.70 (2, 86.59)**	.03	5.36 (.34)	17.29 (2, 152.43)**	.05	4.62 (.20)	43.74 (2, 335.09)**	.06
	FASD+	5.19 (.53)			2.99 (.44)			4.02 (1.49)			5.00 (.44)		
	NDDs	3.04 (.04)			2.70 (.12)			3.22 (.12)			2.81 (.08)		

MANCOVA controlled for age, sex, EFSL status, home problems, and neighbourhood SES. Higher scores represent greater ability

*Indicates *p* ≤ .01

**Indicates* p* ≤ .001

For Manitoba, there were significant group differences on all EDI domain scores (*F*_10, 1316_ = 4.16, *p* < 0.001; Pillai’s trace = 0.061). The univariate analyses revealed significant group differences on all the EDI domain scores except language and cognitive development (Table 5). Pairwise comparisons demonstrated that once again, children with FASD-only scored better in these domains, compared to children with other NDDs. The domain scores for the FASD+ group fell somewhere in the middle in this province as well.

For Alberta, there were also group differences on all EDI domain scores (*F*_10, 1466_ = 5.75, *p* =  < 0.001; Pillai’s trace = 0.076). The univariate analyses revealed group differences on all the EDI domain scores, except for the language and cognitive development domain (Table 5). Pairwise comparisons demonstrated that children with FASD-only scored higher in all domains, except language and cognitive development, compared to children with other NDDs. The FASD+ group fell somewhere in the middle once more.

Finally, for British Columbia, there were group differences on all EDI domain scores (*F*_10, 3028_ = 10.84, *p* =  < 0.001; Pillai’s trace = 0.069). The univariate analyses revealed group differences on four of the five EDI domain scores—all but emotional maturity (Table 5). Children with FASD-only scored higher in these domains compared to children with other NDDs. In addition, children with FASD+ scored higher than their peers with NDDs on the language and cognitive development, and the communication skills and general knowledge domains.

## Discussion

This study adds considerably to the existing literature on FASD in Canada, by providing a population-level, national overview of the prevalence of kindergarten children reported on the EDI as having been diagnosed with FASD, and by providing information on their development, compared to children with other NDDs at school entry in Canada. Across provinces and territories, there was a higher percentage of children diagnosed with other NDDs (0.61–1.92%), compared to those diagnosed with FASD only (0.1–0.22%), or FASD and comorbidities (0–0.1%), to the combined range of 0.1–0.30% (highest in Manitoba). These percentages are less than the recently estimated population prevalence of FASD of 4% in Canada (Flannigan et al. 2018) as well as the only other population-level estimate of prevalence of FASD from a study conducted with children aged 7–9 years using a rigorous epidemiological approach and active case ascertainment, which was 1.8% (Popova et al. 2019), suggesting that identification of this population is occurring at a later age. Thus, at least prior to the emergence of the most recent diagnostic guidelines, FASD may have been significantly under-reported by educators in kindergarten children. This is in keeping with reports that most children are diagnosed later in life, possibly due to developmental trends, symptom overlap, and lack of physiological indicators, and this raises concern about the current ability to detect and consequently respond to the needs presented in young children with FASD (Rasmussen 2005; Rasmussen and Bisanz 2009; Temple et al. 2019). On the other hand, the almost consistently lowest scores among the children with other NDDs likely result from the medical complexities common in that group (Williams et al. 2018).

Analysis of teacher-reported home problems in Canadian children with identified neurological conditions revealed a greater percentage of children with FASD, both with (32.6%) and without comorbidities (25.6%), were identified as having problems at home that interfered with their ability to function in the classroom, compared to children with other NDDs (11.4%). This is consistent with other evidence (Coggins et al. 2007), including evidence that a disproportionate number of children with FASD are placed in foster care compared to the general population (Koponen et al. 2009; Lebel et al. 2019; Price et al. 2017). This finding underscores the vulnerability of this unique group and highlights the need for early identification that may promote increased efforts to enhance stability and implement school-based intervention.

Despite the increased rate of home problems reported in the FASD groups, children with FASD-only generally outperformed their peers with other NDDs across all (or almost all) developmental domains. This may reflect the inability of current assessment tools to detect areas of difficulty within the FASD population, or the unique developmental trajectories for children with FASD characterized by problems compared to those with other NDDs. For example, some higher-level processes that are impacted by FASD, such as executive functioning, develop later in childhood, and thus deficits in these skills tend to become more apparent as children get older, compared to their typically developing peers (Kingdon et al. 2016; Rasmussen et al. 2008). These difficulties may not be observable at such a young age, which could prevent early and appropriate intervention that is linked with better long-term outcomes (Olson et al. 2007). Children who have already been identified with FASD at school entry are not doing as poorly as children with other NDDs but are still showing some developmental difficulties at a young age. Future research, potentially possible through linkage of EDI data with diagnostic information from children’s primary age years, could help establish whether there are differential patterns in children’s developmental health at school entry among those who receive a diagnosis of FASD later than the cohort in our study.

Although the results of the current study reveal some provincial differences, they were minimal, overall revealing a high level of national consistency. Of particular relevance for the diagnosis of FASD, patterns of ‘emotional maturity’ differed by province. In Ontario and British Columbia, there were no differences between the FASD-only and NDD groups, while the FASD-only group had higher scores in Manitoba and Alberta compared to the NDD group. These provincial differences may reflect subtle variations in diagnostic and support practices due to team composition or tools used, regional differences in populations identified, or differences in how diagnostic information is communicated to educators. There could also be jurisdictional differences in access to those services and to professionals who can make the diagnosis, such as doctors and professionals; or in the degree of communication between family, health professionals, and school. Our study was only able to demonstrate the differences in prevalence and outcomes, but not address their causes.

Nevertheless, the small magnitude of differences suggests that Canada in fact is largely consistent in their diagnostic practices (Chudley 2018). This likely reflects the presence of a national diagnostic framework—the only one of its kind in the world used by most FASD clinics in Canada (Chudley et al. 2005). Across Canada, further efforts to accurately identify children with FASD at a young age are underway, aligned with efforts to harmonize service delivery to ensure children with FASD receive consistent and high-quality support (Cook et al. 2016).

### Strengths and Limitations

This study provided a unique teacher-centered perspective on the developmental health of children with FASD, with and without comorbidities, compared to their peers with other NDDs, drawing from a population of over 600,000 children. Our study had a nearly national scope, and thus was able to offer evidence on prevalence among Canadian jurisdictions using the same methodology. Moreover, this study provides a unique insight on how FASD symptoms manifest in kindergarten, a period of time that may not have been adequately explored as the mean age of diagnosis of FASD is 10 years of age (Streissguth et al. 2004). Moreover, although a reliable teacher assessment of children’s abilities is also a strength (in comparison to parental assessment, for example), the inability to follow up with a “case ascertainment” methodology (i.e. accessing existing health services data to confirm the diagnosis) is a limitation of this study. In addition, since the sample sizes of several of the provinces and territories were too small to analyze on their own, inferences from the data are best suited at a national level. In presenting the study, we deemed the children with other NDDs as a better suited control group than typically developing children. Although we believe this decision had strong merit for the purposes of descriptive analyses, since the disparity between developmental status of children with FASD and typically developing population is well documented, the other NDDs group encompassed a multitude of disorders which may have limited their comparability with children with FASD. A future study might employ more complex statistical algorithms, such as propensity score matching, to select a more suitable comparison sample from among the children with other NDDs.

## Conclusion

In this population-level study of kindergarten children in Canada, we reported prevalence of FASD of up to 0.30% among provincial and territorial jurisdictions, with the lowest level in Quebec (0.01%), and highest in Manitoba; all of them lower than the prevalence of other NDDs. The prevalence of FASD in our sample was lower than in two other recent Canadian estimates; but since it is generally diagnosed when children are older, it is possible some deficits are not yet evident in kindergarten. Even though children with FASD only had better outcomes than those with FASD and comorbidities, on average, all kindergarten children with FASD had better developmental health than children with other NDDs at this early age—a finding that may not be generalizable to outcomes in later childhood. Results also demonstrated that a greater proportion of children with FASD, than those with other NDDs, experienced problems at home, in keeping with reports of higher levels of childhood experiences of adversity in this population. This has implications for unique supports that respond to the dual impact of both brain-based differences and environmental vulnerabilities that may combine to impact later outcomes for these children. A better understanding of the characteristics of FASD, especially by educators, may be an important starting point in improving our ability to distinguish features that may help with identifying children earlier in order to facilitate early intervention initiatives and anticipate future needs to support proactive and strengths-based access to support and appropriate services.

## Electronic supplementary material

Below is the link to the electronic supplementary material.

Supplementary file1 (PDF 172 kb)
