# Supplementary material for: Teacher-Reported Prevalence of FASD in Kindergarten in Canada: Association with Child Development and Problems at Home
Source: J Autism Dev Disord. 2020 May 29;51(2):433–43. doi: 10.1007/s10803-020-04545-w (PMC7835185; doi:10.1007/s10803-020-04545-w)
Supplement: Supplementary file 1 — Supplementary file1 (PDF 172 kb) [file 10803_2020_4545_MOESM1_ESM.pdf]

Appendix

Appendix 1. EDI domain scores for all children in Canada

Domain	Mean	Standard Deviation
Physical Health & Well-Being	8.71	1.43
Social Competence	8.21	1.92
Emotional Maturity	7.92	1.59
Language and Cognitive Development	8.42	1.87
Communication Skills and General Knowledge	7.61	2.65

Note. EDI domain scores range from 0 to 10

Appendix 2. EDI subdomain scores for all children in Canada

Subdomains	Mean	Standard Deviation
Physical Health & Well-Being subdomains		
Physical Readiness for School Delay subdomain	8.79	2.11
Physical Independence subdomain	9.64	1.12
Gross and Fine Motor Skills subdomain	7.90	2.41
Social Competence subdomains		
Overall social competence subdomain	7.66	2.45
Responsibility and Respect subdomain	8.47	2.11
Approaches to Learning subdomain	8.00	2.26
Readiness to Explore New Things subdomain	8.87	1.95
Emotional Maturity subdomains		
Prosocial and Helping Behaviour subdomain	5.57	3.07
Anxious and Fearful Behaviour subdomain	8.86	1.55
Aggressive Behaviour subdomain	9.17	1.58
Hyperactivity and Inattentive Behaviour subdomain	8.18	2.51
Language and Cognitive Development subdomains		
Basic Literacy subdomain	9.12	1.75
Interest Literacy/Numeracy and Memory subdomain	8.92	2.24
Advanced Literacy subdomain	6.16	3.27
Basic Numeracy subdomain	9.21	1.87

Note. The communication skills and general knowledge subdomain is the same as the domain and therefore is not included in this table. EDI subdomain scores range from 0 to 10

Appendix 3. MANCOVA univariate results examining differences in scores on all 16 subdomains in Canada among children with FASD-only, children with FASD and other comorbidities, and children with other NDDs

		Mean	Standard Error	F	*p*
Physical readiness for the school day	FASD only	7.56	0.12	F (2, 37.28) = 5.44	.004
	FASD+	7.46	0.24		
	NDDs	7.90	0.03		
Physical independence	FASD only	8.46	0.12	F (2, 408.12) = 54.11	< .001
	FASD+	7.75	0.25		
	NDDs	7.15	0.03		
Gross and fine motor skills	FASD only	5.62	0.13	F (2, 262.30) = 32.84	< .001
	FASD+	5.30	0.26		
	NDDs	4.59	0.03		
Overall social competence	FASD only	4.37	0.12	F (2, 182.43) = 27.60	< .001
	FASD+	3.59	0.23		
	NDDs	3.48	0.03		
Responsibility and respect	FASD only	5.65	0.13	F (2, 47.718) = 6.01	.002
	FASD+	4.93	0.26		
	NDDs	5.22	0.03		
Approaches to learning	FASD only	4.73	0.11	F (2, 149.38) = 23.77	< .001
	FASD+	4.29	0.23		
	NDDs	3.93	0.03		
Readiness to explore new things	FASD only	7.83	0.14	F (2, 525.31) = 58.90	< .001
	FASD+	7.13	0.27		
	NDDs	6.35	0.03		
Prosocial and helping behaviour	FASD only	3.44	0.11	F (2, 613.58) = 108.32	< .001
	FASD+	3.01	0.22		
	NDDs	1.88	0.03		
Anxious and fearful behaviour	FASD only	7.85	0.11	F (2, 10.59) = 2.83	.059
	FASD+	7.39	0.22		
	NDDs	7.71	0.03		
Aggressive behaviour	FASD only	7.13	0.09	F (2, 155.74) = 33.98	< .001
	FASD+	7.08	0.18		
	NDDs	7.86	0.02		
Hyperactivity and inattentive behaviour	FASD only	4.19	0.10	F (2, 35.29) = 3.75	.024
	FASD+	4.00	0.19		
	NDDs	4.50	0.03		
Basic literacy	FASD only	7.09	0.14	F (2, 146.21) = 13.85	<.001
	FASD+	6.73	0.28		
	NDDs	6.31	0.04		
Interest in literacy/numeracy and memory	FASD only	7.14	0.15	F (2, 171.36) = 14.22	< .001
	FASD+	6.69	0.29		
	NDDs	6.29	0.04		
Advanced literacy	FASD only	3.29	0.16	F (2, 1.41) = 0.12	.890
	FASD+	3.38	0.31		
	NDDs	3.37	0.04		
Basic numeracy	FASD only	6.96	0.16	F (2, 104.90) = 8.05	< .001
	FASD+	6.89	0.32		

	NDDs	6.34	0.04		
Communication skills and general knowledge	FASD only	4.75	0.13	$F(2, 585.07) = 69.98$	< .001
	FASD+	4.17	0.26		
	NDDs	3.21	0.03		

Note. MANCOVA controlled for age, sex, EFSL status, home problems, and neighbourhood SES.

Appendix 4. MANCOVA univariate results examining differences in scores on all 16 subdomains in Ontario among children with FASD-only, children with FASD and other comorbidities, and children with other NDDs

		Mean	Standard Error	F	p
Physical readiness for the school day	FASD only	7.26	.28	F (2, 7.16) = 2.48	.625
	FASD+	7.73	.52		
	NDDs	7.88	.04		
Physical independence	FASD only	8.56	.29	F (2, 77.82) = 10.41	< .001
	FASD+	7.71	.54		
	NDDs	7.25	.04		
Gross and fine motor skills	FASD only	5.99	.29	F (2, 109.68) = 14.20	< .001
	FASD+	6.28	.55		
	NDDs	4.67	.05		
Overall social competence	FASD only	4.22	.26	F (2, 36.73) = 5.88	.003
	FASD+	3.66	.49		
	NDDs	3.32	.04		
Responsibility and respect	FASD only	5.24	.30	F (2, 4.04) = 0.51	.598
	FASD+	4.63	.55		
	NDDs	5.04	.05		
Approaches to learning	FASD only	4.59	.26	F (2, 23.69) = 3.96	.019
	FASD+	4.43	.48		
	NDDs	3.90	.04		
Readiness to explore new things	FASD only	7.73	.32	F (2, 111.69) = 12.13	< .001
	FASD+	6.65	.60		
	NDDs	6.14	.05		
Prosocial and helping behaviour	FASD only	3.54	.25	F (2, 159.98) = 29.50	< .001
	FASD+	3.69	.46		
	NDDs	1.88	.04		
Anxious and fearful behaviour	FASD only	7.71	.21	F (2, 2.65) = 0.68	.505
	FASD+	7.23	.39		
	NDDs	7.68	.03		
Aggressive behaviour	FASD only	6.38	.24	F (2, 92.07) = 18.33	< .001
	FASD+	6.40	.44		
	NDDs	7.67	.04		
Hyperactivity and inattentive behaviour	FASD only	3.88	.32	F (2, 19.34) = 2.07	.127
	FASD+	3.70	.60		
	NDDs	4.42	.05		
Basic literacy	FASD only	7.61	.34	F (2, 75.29) = 7.25	.001
	FASD+	7.60	.63		
	NDDs	6.45	.05		
Interest in literacy/numeracy and memory	FASD only	7.30	.38	F (2, 52.11) = 4.14	.016
	FASD+	6.35	.70		
	NDDs	6.21	.06		
Advanced literacy	FASD only	4.17	.38	F (2, 22.75) = 1.79	.167
	FASD+	4.31	.70		
	NDDs	3.56	.06		
Basic numeracy	FASD only	7.64	.38	F (2, 58.84) = 4.68	.009
	FASD+	7.74	.70		

	NDDs	6.39	.06		
Communication skills and general knowledge	FASD only	5.09	.30	F (2, 222.54) = 28.08	< .001
	FASD+	5.15	.55		
	NDDs	3.11	.05		

Note. MANCOVA controlled for age, sex, EFSL status, home problems, and neighbourhood SES.

Appendix 5. MANCOVA univariate results examining differences in scores on all 16 subdomains in Manitoba among children with FASD-only, children with FASD and other comorbidities, and children with other NDDs

		Mean	Standard Error	F	*p*
Physical readiness for the school day	FASD only	8.00	.30	F (2, 5.51) = .853	.427
	FASD+	7.45	.42		
	NDDs	8.02	.11		
Physical independence	FASD only	8.47	.32	F (2, 149.66) = 19.54	< .001
	FASD+	7.57	.46		
	NDDs	6.40	.12		
Gross and fine motor skills	FASD only	5.77	.32	F (2, 101.96) = 13.18	< .001
	FASD+	4.93	.46		
	NDDs	4.05	.12		
Overall social competence	FASD only	4.76	.30	F (2, 64.61) = 9.79	< .001
	FASD+	3.37	.43		
	NDDs	3.34	.11		
Responsibility and respect	FASD only	5.93	.32	F (2, 20.74) = 2.73	.066
	FASD+	5.23	.46		
	NDDs	5.12	.12		
Approaches to learning	FASD only	4.79	.29	F (2, 55.22) = 9.04	< .001
	FASD+	4.26	.41		
	NDDs	3.53	.11		
Readiness to explore new things	FASD only	8.20	.34	F (2, 118.33) = 14.12	< .001
	FASD+	7.23	.48		
	NDDs	6.34	.13		
Prosocial and helping behaviour	FASD only	3.07	.28	F (2, 71.71) = 12.60	< .001
	FASD+	2.83	.40		
	NDDs	1.72	.11		
Anxious and fearful behaviour	FASD only	8.00	.22	F (2, 6.59) = 1.79	.168
	FASD+	7.30	.32		
	NDDs	7.66	.09		
Aggressive behaviour	FASD only	7.52	.25	F (2, 1.39) = 0.29	.747
	FASD+	7.76	.36		
	NDDs	7.72	.10		
Hyperactivity and inattentive behaviour	FASD only	4.65	.37	F (2, 12.00) = 1.17	.310
	FASD+	4.40	.53		
	NDDs	4.07	.14		
Basic literacy	FASD only	6.60	.37	F (2, 41.89) = 4.10	.017
	FASD+	5.51	.53		
	NDDs	5.46	.14		
Interest in literacy/numeracy and memory	FASD only	6.80	.39	F (2, 31.60) = 2.84	.059
	FASD+	5.74	.55		
	NDDs	5.82	.15		
Advanced literacy	FASD only	2.74	.37	F (2, 2.16) = 0.21	.810
	FASD+	2.37	.53		
	NDDs	2.51	.14		
Basic numeracy	FASD only	6.70	.42	F (2, 75.60) = 5.74	.003
	FASD+	5.45	.60		
	NDDs	5.16	.16		

Communication skills and general knowledge	FASD only	4.34	.32	F (2, 80.71) = 10.59	< .001
	FASD+	3.00	.46		
	NDDs	2.75	.12		

Note. MANCOVA controlled for age, sex, EFSL status, home problems, and neighbourhood SES.

Appendix 6. MANCOVA univariate results examining differences in scores on all 16 subdomains in Alberta among children with FASD-only, children with FASD and other comorbidities, and children with other NDDs

		Mean	Standard Error	F	*p*
Physical readiness for the school day	FASD only	8.31	.31	F (2, 3.29) = .471	.625
	FASD+	7.82	1.33		
	NDDs	8.00	.11		
Physical independence	FASD only	9.09	.32	F (2, 142.90) = 19.27	< .001
	FASD+	8.90	1.37		
	NDDs	7.04	.11		
Gross and fine motor skills	FASD only	6.08	.33	F (2, 105.15) = 13.46	< .001
	FASD+	6.03	1.40		
	NDDs	4.33	.11		
Overall social competence	FASD only	4.73	.30	F (2, 61.28) = 9.20	< .001
	FASD+	4.45	1.30		
	NDDs	3.38	.10		
Responsibility and respect	FASD only	6.13	.34	F (2, 33.62) = 4.05	.018
	FASD+	6.60	1.45		
	NDDs	5.17	.12		
Approaches to learning	FASD only	5.30	.30	F (2, 57.90) = 8.73	< .001
	FASD+	3.86	1.29		
	NDDs	3.97	.10		
Readiness to explore new things	FASD only	8.25	.35	F (2, 106.70) = 12.05	< .001
	FASD+	5.63	1.49		
	NDDs	6.47	.12		
Prosocial and helping behaviour	FASD only	4.18	.29	F (2, 190.83) = 31.76	< .001
	FASD+	4.44	1.23		
	NDDs	1.83	.10		
Anxious and fearful behaviour	FASD only	8.09	.22	F (2, 3.71) = 1.06	.348
	FASD+	8.32	.94		
	NDDs	7.77	.08		
Aggressive behaviour	FASD only	7.64	.22	F (2, 11.61) = 3.30	.038
	FASD+	9.42	.94		
	NDDs	8.14	.08		
Hyperactivity and inattentive behaviour	FASD only	4.69	.35	F (2, 3.45) = 0.38	.687
	FASD+	3.35	1.52		
	NDDs	4.67	.12		
Basic literacy	FASD only	7.30	.38	F (2, 38.35) = 3.67	.026
	FASD+	7.41	1.62		
	NDDs	6.25	.13		
Interest in literacy/numeracy and memory	FASD only	7.47	.41	F (2, 48.83) = 4.04	.018
	FASD+	7.12	1.74		
	NDDs	6.26	.14		
Advanced literacy	FASD only	3.21	.38	F (2, 3.99) = 0.37	.692
	FASD+	4.40	1.65		
	NDDs	3.07	.13		
Basic numeracy	FASD only	7.01	.42	F (2, 35.14) = 2.66	.071
	FASD+	7.60	1.83		
	NDDs	6.04	.15		
Communication skills and general knowledge	FASD only	5.27	.35	F (2, 135.21) = 15.10	< .001
	FASD+	3.70	1.50		
	NDDs	3.24	.12		

Note. MANCOVA controlled for age, sex, EFSL status, home problems, and neighbourhood SES.

Appendix 7. MANCOVA univariate results examining differences in scores on all 16 subdomains in British Columbia among children with FASD-only, children with FASD and other comorbidities, and children with other NDDs

		Mean	Standard Error	F	*p*
Physical readiness for the school day	FASD only	7.20	.20	F (2, 7.94) = 1.11	.331
	FASD+	7.08	.43		
	NDDs	7.47	.08		
Physical independence	FASD only	8.28	.21	F (2, 208.30) = 26.36	< .001
	FASD+	8.12	.45		
	NDDs	6.74	.08		
Gross and fine motor skills	FASD only	5.23	.21	F (2, 157.59) = 20.71	< .001
	FASD+	5.42	.44		
	NDDs	3.95	.08		
Overall social competence	FASD only	4.08	.19	F (2, 50.50) = 8.13	< .001
	FASD+	3.76	.40		
	NDDs	3.29	.07		
Responsibility and respect	FASD only	5.44	.21	F (2, 15.57) = 1.91	.148
	FASD+	4.76	.46		
	NDDs	5.02	.08		
Approaches to learning	FASD only	4.52	.19	F (2, 64.13) = 9.69	< .001
	FASD+	4.39	.41		
	NDDs	3.66	.08		
Readiness to explore new things	FASD only	7.62	.21	F (2, 121.30) = 14.95	< .001
	FASD+	7.99	.46		
	NDDs	6.54	.08		
Prosocial and helping behaviour	FASD only	3.37	.17	F (2, 209.50) = 38.35	< .001
	FASD+	2.73	.38		
	NDDs	1.77	.07		
Anxious and fearful behaviour	FASD only	7.58	.15	F (2, 0.45) = 0.11	.893
	FASD+	7.49	.32		
	NDDs	7.62	.06		
Aggressive behaviour	FASD only	6.99	.16	F (2, 91.12) = 20.45	< .001
	FASD+	6.51	.34		
	NDDs	7.88	.06		
Hyperactivity and inattentive behaviour	FASD only	3.91	.23	F (2, 23.37) = 2.41	.090
	FASD+	3.88	.50		
	NDDs	4.41	.09		
Basic literacy	FASD only	7.07	.25	F (2, 122.23) = 10.72	< .001
	FASD+	7.31	.54		
	NDDs	5.96	.10		
Interest in literacy/numeracy and memory	FASD only	7.17	.25	F (2, 78.69) = 6.87	.001
	FASD+	7.73	.54		
	NDDs	6.37	.10		
Advanced literacy	FASD only	2.99	.25	F (2, 6.58) = 0.57	.567
	FASD+	3.57	.54		
	NDDs	2.98	.10		
Basic numeracy	FASD only	6.90	.28	F (2, 166.51) = 11.85	< .001
	FASD+	7.91	.60		
	NDDs	5.79	.11		
Communication skills and general knowledge	FASD only	4.56	.21	F (2, 280.90) = 35.85	< .001
	FASD+	4.95	.45		
	NDDs	2.88	.08		

Note. MANCOVA controlled for age, sex, EFSL status, home problems, and neighbourhood SES.
